# Performance of the Predicting Risk of Cardiovascular Disease Events Calculator in Rheumatoid Arthritis

**DOI:** 10.1002/art.70081

**Published:** 2026-02-17

**Authors:** Tate M. Johnson, Halie Frideres, Punyasha Roul, Joshua F. Baker, Brian C. Sauer, Grant W. Cannon, Isaac D. Smith, Gary A. Kunkel, Beth I. Wallace, Thomas R. Porter, Kaveh R. Bookani, Amarnath R. Annapureddy, Ted R. Mikuls, Bryant R. England

**Affiliations:** 1 Veterans Affairs (VA) Nebraska-Western Iowa Health Care System, Omaha, Nebraska; 2 Division of Rheumatology & Immunology, University of Nebraska Medical Center, Omaha; 3 Corporal Michael J. Crescenz VA Medical Center, University of Pennsylvania, Philadelphia; 4 Salt Lake City VA Health Care, University of Utah, Salt Lake City; 5 Durham VA Health Care System, Durham, North Carolina; 6 VA Ann Arbor Center for Clinical Management Research, Ann Arbor, Michigan; 7 Division of Rheumatology, University of Michigan, Ann Arbor; 8 Division of Cardiovascular Medicine, University of Nebraska Medical Center, Omaha.

## Abstract

**Objective.:**

Evaluate performance of the Predicting Risk of Cardiovascular Disease Events (PREVENT) calculator in rheumatoid arthritis (RA).

**Methods.:**

Patients with RA were matched up to 10 controls on age, sex, and enrollment year using National Veterans Health Administration, Medicare, and National Death Index data (2006–2020). Ten-year estimated cardiovascular disease (CVD) risk was calculated using PREVENT. Calibration (standardized incidence ratio [SIR] and observed/predicted events) and discrimination (sensitivity and Harrel’s C-statistics) were compared between RA cases and controls. PREVENT performance was compared with the Pooled Cohort Equations (PCE) for atherosclerotic CVD (ASCVD) prediction in RA, including net reclassification index (NRI) calculation.

**Results.:**

Among 30,687 patients with RA and 231,752 patients without RA, 28,061 ASCVD and 13,851 heart failure (HF) outcomes were identified over >2 million person-years. PREVENT underestimated overall CVD (SIR 1.83, 95% confidence interval [CI] 1.79–1.88), ASCVD (SIR 2.25, 95% CI 2.19–2.32) and HF risk (SIR 1.41, 95% CI 1.36–1.46) to a greater degree in RA compared with controls and exhibited poor sensitivity for ASCVD (61.9%) and HF (63.2%) development. PREVENT performance was poorer for ASCVD prediction compared with the PCE (SIR 1.38, 95% CI 1.34–1.41, sensitivity 76.0%). NRI for PREVENT was modest (5.3%). Among 657 reclassified patients who experienced ASCVD, 626 were inappropriately reclassified as low or borderline risk. PREVENT performance significantly improved when including hemoglobin A1c (overall CVD: SIR 1.21, 95% CI 1.18–1.24; ASCVD: SIR 1.45, 95% CI 1.41–1.50; HF: SIR 0.79, 95% CI 0.76–0.82; sensitivity ASCVD 80.3%).

**Conclusion.:**

PREVENT underestimates CVD risk in RA, consistent with suboptimal performance of existing risk calculators. Preferential use of PREVENT including hemoglobin A1c should be considered. Improving CVD risk stratification in RA remains a high priority.

## INTRODUCTION

Rheumatoid arthritis (RA) is an autoimmune inflammatory arthritis with extra-articular manifestations that lead to premature death.^1,2^ Among extra-articular manifestations, cardiovascular disease (CVD) is the most frequent cause of death in patients with RA and accounts for the majority of excess deaths in this high-risk population.^3^ Existing general population CVD risk calculators typically underestimate CVD risk in patients with RA, including those incorporating only traditional CVD risk factors^4–6^ and those that account for systemic inflammation.^4^ Several efforts have been made to develop RA-specific risk calculators, including the Expanded Cardiovascular Risk Prediction Score for RA (accounting for RA duration, disease activity, disability, and prednisone use)^7^ and the EULAR 1.5× multiplier (accounting for seropositivity, extra-articular disease, and disease duration).^8^ However, the EULAR multiplier was not based on direct evidence,^8^ and neither outperforms general population calculators in external validation studies.^9^ A multibiomarker disease activity (MBDA)–based CVD risk score, including the MBDA score, leptin, matrix metalloproteinase-3, and TNF receptor 1 concentrations along-side several clinical variables, strongly associates with three-year CVD risk, although its performance has not been tested against routinely available general population risk calculators.^10,11^ Taken together, there is currently no data-driven strategy for CVD risk stratification in RA.

The American Heart Association (AHA) recently developed the Predicting Risk of CVD Events (PREVENT) calculators as an update to the Pooled Cohort Equations (PCE) currently used for CVD risk stratification in the general population.^12^ PREVENT was developed with recognition of cardiovascular–kidney–metabolic syndrome, accounting for the interplay between chronic kidney disease, metabolic comorbidity (eg, obesity and diabetes), and CVD risk.^13^ It newly incorporates body mass index (BMI) and renal function, as well as the option to include diabetes severity using hemoglobin A1c (HBA1c). Additional updates include removal of race and optional inclusion of the Area Deprivation Index (ADI), a composite measure quantifying a geographic region’s socioeconomic deprivation.^14^ Recognizing the substantial burden of heart failure (HF) on patients and health care systems in the United States,^15^ PREVENT also includes the option to estimate HF risk in addition to atherosclerotic CVD (ASCVD), contrasting the PCE. Given the lack of an accepted CVD risk stratification strategy in RA, an AHA-recognized CVD risk-enhancing factor,^16^ as well as limited existing external validation of PREVENT, the objectives of this study were to (1) evaluate performance of PREVENT in patients with RA compared with matched controls and (2) compare performance of PREVENT to that of the PCE in patients with RA.

## PATIENTS AND METHODS

### Database and study population.

This study was conducted using National Veterans Health Administration (VHA) data between January 1, 2006, and December 31, 2019. The VHA is the largest integrated health system in the United States, with detailed administrative and electronic health record data, including demographics, vital signs, diagnostic and procedural codes from inpatient and outpatient care, laboratory values, and pharmacy dispensations. Data are queried from the Corporate Data Warehouse (CDW) within the Veterans Affairs (VA) Informatics and Computing Infrastructure (VINCI).^17^ Capture of CVD events outside of the VHA was accomplished through query of fee-for-service charges and Center for Medicare and Medicaid Services (CMS) linkage. These databases were also linked to the National Death Index (NDI) for assessment of vital status and underlying causes of death. This study received institutional review board approval through the VA Nebraska-Western Iowa Healthcare System and the University of Utah.

We constructed a retrospective, matched cohort of patients with RA, requiring the presence of two or more *International Classification of Diseases, Ninth Edition* (*ICD-9*) or *International Classification of Diseases, Tenth Edition* (*ICD-10*) diagnostic codes for RA, a rheumatologist diagnosis of RA, plus either a positive RA autoantibody (rheumatoid factor [RF] or anti–cyclic citrullinated protein [anti-CCP] antibody) or dispensing of a disease-modifying antirheumatic drug during the study period.^18^ RA cases were matched with up to 10 non-RA controls within the VHA system on year of birth, sex, and VHA enrollment year. Cohort entry was the date of RA diagnosis for cases and the corresponding calendar date for controls. Eligible patients were aged 30 to 79 years and free of ASCVD or HF before cohort entry. Patients were followed for up to 10 years from cohort entry to the earliest of an incident CVD event, death from a non-CVD–related cause, or end of study period.

### Cardiovascular risk calculation.

Variables included in the PREVENT and PCE calculators (Supplementary Table 1) were assessed at baseline, restricting to a 12-month lookback period to more accurately reflect current comorbidities and medication use. Demographics (age, sex, and self-reported race) were obtained from VHA enrollment records. Vital signs from the most recent VHA encounter were used to record BMI and blood pressure (BP). Self-reported smoking status was retrieved from VHA encounters.^19^ Diabetes was defined by the presence of two or more diagnostic codes for diabetes occurring at least 30 days apart or a HBA1c measurement ≥6.5%. Antihypertensive, aspirin, and lipid-lowering therapy use were defined by a pharmacy-dispensing episode during the lookback period. Serum lipid levels and creatinine were extracted from laboratory data within CDW. Estimated glomerular filtration rate (eGFR) was then calculated using the Chronic Kidney Disease Epidemiology Collaboration Creatinine Equation (2021). ADI was defined using zip code linkage between patients’ recorded residences and the 2020 version of publicly available ADI data.^20^

Missing risk calculator variables were imputed by multiple imputations with chained equations with 10 imputations, using the average imputed risk score for each patient. Patients with values falling outside valid ranges for PREVENT or PCE risk calculation were excluded. Estimated 10-year probability of a CVD event was determined for each patient using the log-odds resulting from published regression equations for PREVENT and PCE calculators.^12,21^ For those with less than 10 years of follow-up who did not experience a CVD event, the person-time denominator in determining the expected event probability was reduced proportionally.^6^

### Cardiovascular outcome assessment.

CVD outcomes reflect those that PREVENT was developed to predict, including an overall CVD composite (fatal and nonfatal acute myocardial infarction [AMI], stroke, or HF), ASCVD (AMI or stroke), and HF. Nonfatal CVD events were identified using *ICD-9* and *ICD-10* diagnostic and procedure codes requiring a primary or secondary discharge diagnosis for AMI or stroke that have been validated in prior analyses of ASCVD risk in the VA.^22–24^ A primary discharge diagnosis was required to define a HF event to maximize positive predictive value for incident HF.^22,25^ Date of death was determined through NDI linkage, identifying causes of CVD-related death using *ICD-10* categories defined by the Clinical Classifications Software Refined through the Healthcare Cost and Utilization Project.^3,26^

### Statistical analysis.

Baseline characteristics of the cohort were described, and incidence rates for individual and composite CVD events stratified by RA status were calculated. For both PREVENT and PCE, we assessed model calibration by calculating standardized incidence ratios (SIR; observed:predicted CVD event rate) with 95% confidence intervals (CIs), assuming a Poisson distribution for observed event rates and that predicted event rates remain fixed.^27^ Calibration was then illustrated across predicted CVD risk values in patients with RA with calibration plots, using the *calibrationbelt* command in Stata. Model discrimination was assessed by calculating Harrel’s C-statistics and sensitivity using a 7.5% risk cutoff (ie, low and borderline vs intermediate and high risk). In secondary analyses, we evaluated performance of PREVENT when incorporating the ADI and HBA1c, then conducted stratified analyses by sex, seropositivity (RF and/or anti-CCP), and comorbid diabetes. In sensitivity analyses, we restricted to patients ≥65 years old (a Medicare-aged cohort with near-complete capture of CVD events) and conducted a complete case analysis, excluding individuals with missing risk calculator components. Lastly, we explored calibration of PREVENT across BMI categories and quintiles of serum low-density lipoprotein (LDL) levels among patients with RA. This exploratory analysis was pursued because (1) BMI is not included in the PREVENT overall and ASCVD regression models and (2) given well described lipid and obesity paradoxes (ie, U-shaped relationships), linear modeling of BMI and LDL in PREVENT was hypothesized to reduce predictive performance at extreme BMI and LDL values in RA.^28,29^

To compare the PREVENT and PCE calculators for predicting ASCVD among patients with RA, PCE performance was assessed as above and compared with PREVENT. We additionally calculated the net reclassification index (NRI) of PREVENT relative to the PCE. The NRI is a calculator’s ability to appropriately recategorize a patient from a lower to higher predicted CVD risk category (or vice versa). The NRI was calculated using a cutoff point of 7.5% and 100 bootstrapped samples for SE estimation. All analyses were completed using StataMP (version 18) and SAS 9.x within VINCI.

### Data availability.

Data are available upon reasonable request and after required administrative approvals are obtained.

## RESULTS

### Baseline characteristics of study cohort.

There were 30,687 patients with RA (mean age 60.9, 84.9% men) and 231,752 matched controls (mean age 59.6, 83.2% men) eligible for inclusion in this study (Table 1). The most commonly reported race was White (75.4% in RA, 63.1% in controls), which was more frequently missing among controls. Consistent with known epidemiologic associations, a greater proportion of patients with RA were ever-smokers (83.0% vs 65.0%) and carried diagnoses of hypertension (51.2% vs 35.3%) or diabetes (22.4% vs 15.7%). A greater proportion of patients with RA were on a lipid-lowering agent (38.4% vs 24.3%), antihypertensive (55.6% in RA vs 32.0% in controls), or aspirin (10.6% vs 6.2%) at baseline. Mean baseline cholesterol, BP, and eGFR were similar. Using PREVENT, a greater proportion of patients with RA were at intermediate or high estimated CVD risk (overall CVD 60.5%, ASCVD 32.6%, HF 32.1% in RA; overall CVD 50.4%, ASCVD 24.5%, HF 23.3% in controls).

### CVD event rates.

Over 2,063,536 person-years (PY) of follow-up (mean follow-up 7.5 [SD 2.7] years in RA, 7.8 [SD 2.5] years in controls), 36,849 overall CVD events occurred (Supplementary Table 2). The incidence of overall CVD (24.9 events per 1,000 PY in RA, 95% CI 24.3–25.6; 16.2 in controls, 95% CI 16.0–16.4), ASCVD (17.8 in RA, 95% CI 17.3–18.4; 12.5 in controls, 95% CI 12.3–12.7), and HF (11.5 in RA, 95% CI 11.1–12.0; 5.8 in controls, 95% CI 5.7–5.9) was higher in RA compared with controls.

### PREVENT performance in RA.

**Figure 1.**
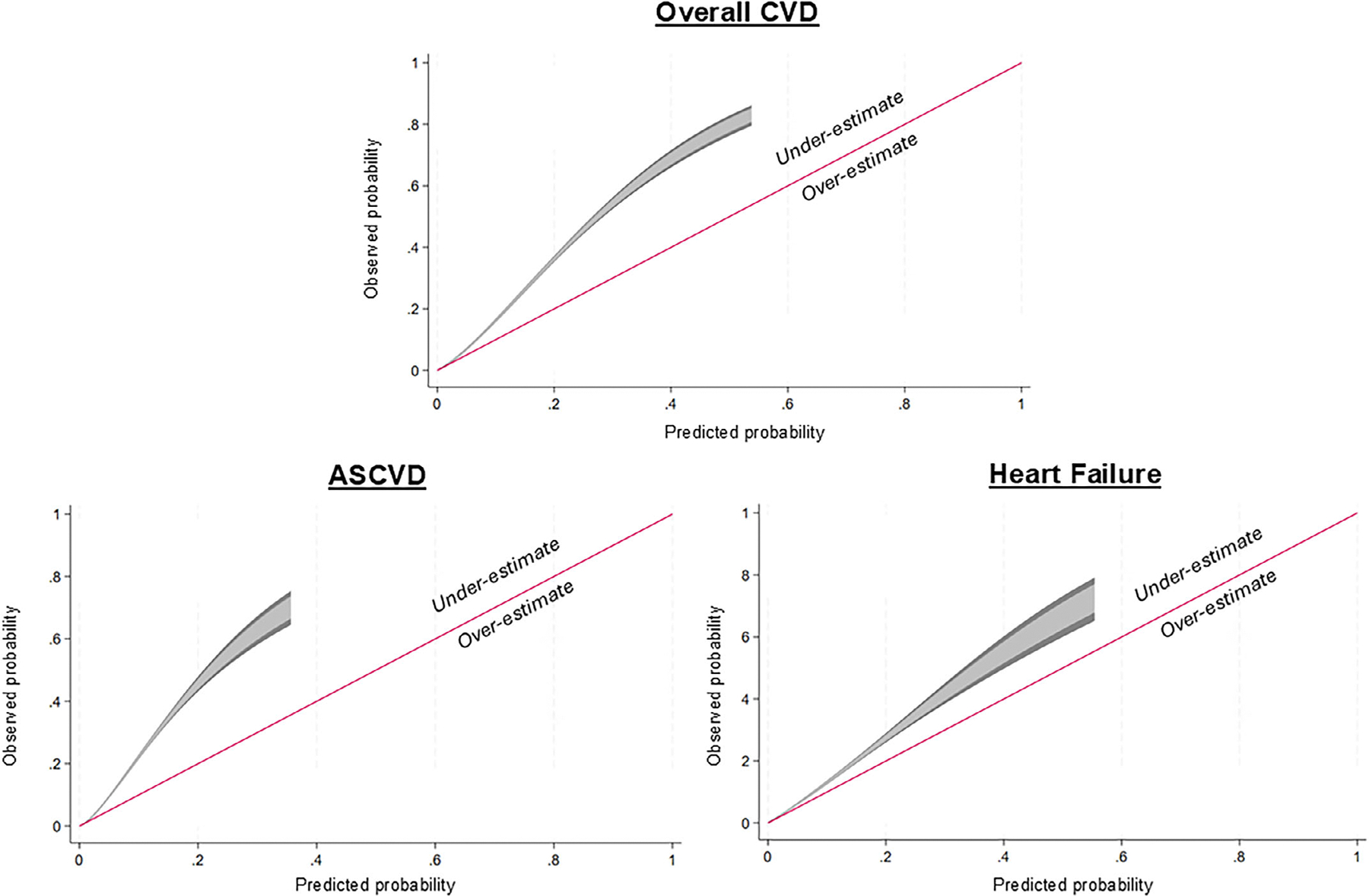
Calibration plot illustrating goodness of fit for the predicted probability of a CVD event using the Predicting Risk in Cardiovascular Disease Events risk calculator in rheumatoid arthritis compared with the observed event probability. The straight line illustrates perfect fit. Deviation above this line represents underestimation of CVD risk (observed probability > predicted probability), whereas deviation under this line represents overestimation of CVD risk. ASCVD, atherosclerotic cardiovascular disease; CVD, cardiovascular disease.

PREVENT underestimated CVD risk (SIR 1.83, 95% CI 1.79–1.88) to a greater degree in RA than among matched controls (SIR 1.44, 95% CI 1.43–1.46) (Table 2). This pattern was similar, although the SIR was greater, for ASCVD (SIR 2.25 in RA, 95% CI 2.19–2.32; SIR 1.86 in controls, 95% CI 1.84–1.89). For HF prediction, calibration improved among controls (SIR 0.89, 95% CI 0.87–0.90), but risk in RA was still underestimated (SIR 1.41, 95% CI 1.36–1.46). Results were similar when stratified by seropositivity of RA cases (Supplementary Table 3), in sensitivity analyses restricting to age ≥65 years, and in complete case analyses (Supplementary Table 4). When stratified by sex, CVD risk was similarly underestimated to a greater degree in RA compared with matched controls, although the magnitude of underestimation was attenuated among women with RA (Supplementary Table 5). In patients with RA, PREVENT underestimated event rates across all levels of predicted CVD risk (Figure 1). Incorporation of the ADI produced similar estimates for overall CVD and ASCVD, whereas calibration worsened for HF prediction in RA (SIR 1.88, 95% CI 1.81–1.95; Table 2). In contrast, incorporation of HBA1c significantly improved calibration for all CVD outcomes in patients with RA regardless of diabetes status (Overall CVD: SIR 1.21, 95% CI 1.18–1.24; ASCVD: SIR 1.45, 95% CI 1.41–1.50; HF: SIR 0.79, 95% CI 0.76–0.82), whereas calibration only modestly improved among patients with a diagnosis of diabetes (Supplementary Table 6).

When using a risk cutoff of 7.5%, PREVENT exhibited good sensitivity for overall CVD in patients with (85.7%) and without (83.2%) RA (Table 2). Sensitivity was reduced when using ASCVD-specific (61.9% RA, 55.2% controls) and HF-specific (63.2% RA, 57.4% controls) risk calculators. After incorporating ADI, sensitivity for overall CVD and ASCVD in RA was similar but worsened for HF (45.3%). Sensitivity improved for all CVD outcomes in RA when including HBA1c (overall CVD 93.1%, ASCVD 80.3%, HF 79.9%) and among those with a diabetes diagnosis (overall CVD 95.0%, ASCVD 85.6%, HF 89.5%; Supplementary Table 6).

The overall discriminatory performance of all calculators was modest (Harrel’s C-statistic 0.66–0.71), regardless of RA status, CVD outcome, or inclusion of ADI or HBA1c. Discrimination was similar (Harrel’s C-statistic 0.59–0.72) when stratifying by seropositivity, restricting to those age ≥65 years and in analyses requiring nonmissing risk calculator components (Supplementary Tables 3 and 4). Restricting to age ≥65 years improved sensitivity for overall CVD (96.9% RA, 98.0% controls), ASCVD (85.8% RA, 80.6% controls), and HF (90.0% RA, 86.2% controls) (Supplementary Table 4).

**Figure 2.**
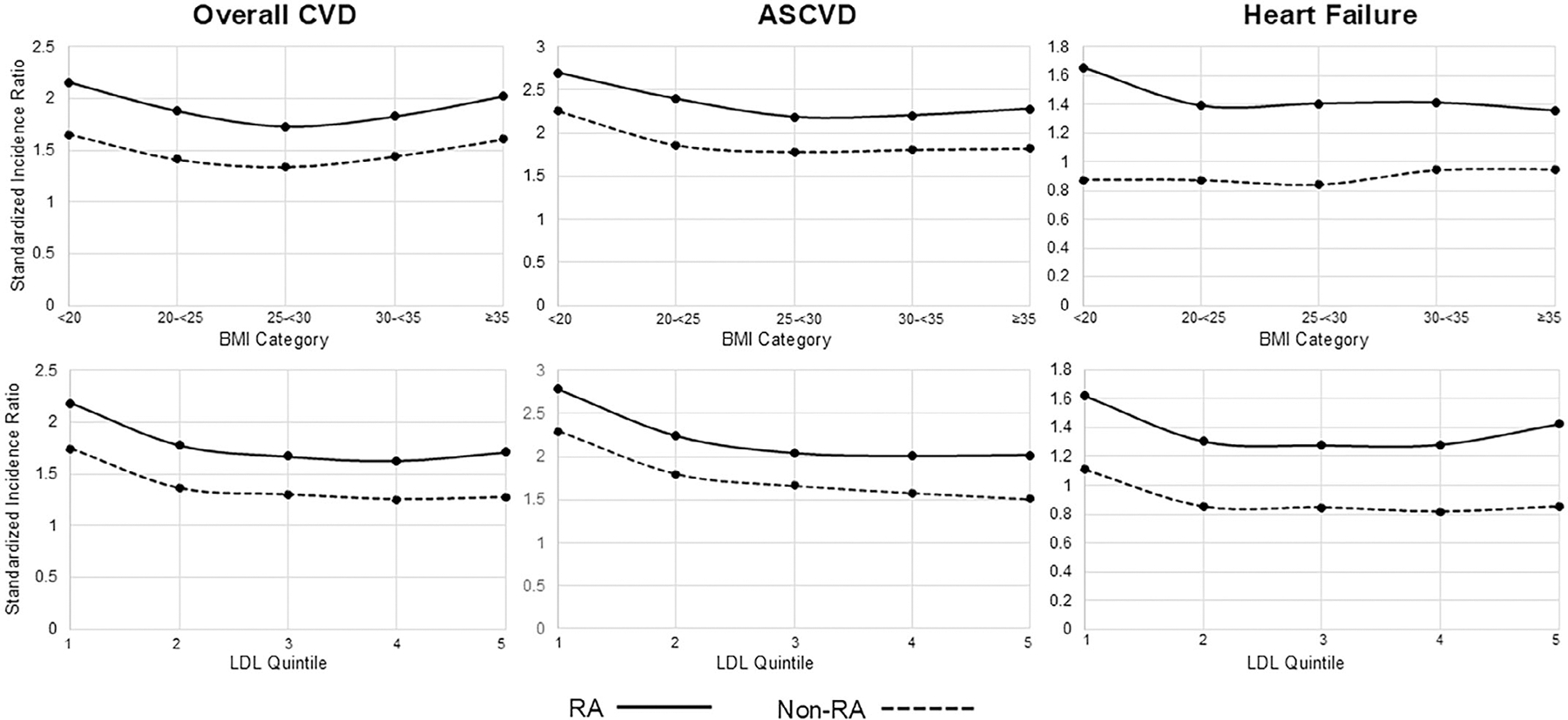
Standardized incidence ratio (observed/predicted CVD event rate) of the Predicting Risk in Cardiovascular Disease Events risk calculator in RA and matched non-RA controls, stratified by BMI categories (top row) and LDL quintiles (bottom row). Quintile 1 represents the lowest LDL values, whereas quintile 5 represents the highest LDL values. ASCVD, atherosclerotic cardiovascular disease; BMI, body mass index; CVD, cardiovascular disease; LDL, low-density lipoprotein; RA, rheumatoid arthritis.

When using PREVENT, the SIR for overall CVD risk in RA exhibited a U-shaped relationship with BMI, with the highest SIR observed among those with the lowest (<20 kg/m^2^, SIR 2.15) and highest (≥35 kg/m^2^, SIR 2.02) BMI (Figure 2). The SIR was highest among patients with low BMI (<20 kg/m^2^) for models estimating ASCVD (SIR 2.70) and HF (SIR 1.66) risk. When stratified by LDL quintiles, the SIR was highest among those with the lowest LDL values for overall CVD (SIR 2.18) and ASCVD (SIR 2.79), whereas a U-shaped relationship between SIR and LDL categories was observed for HF-specific calculators (LDL quintile 1 SIR 1.62; quintile 5 SIR 1.43).

### PREVENT performance vs PCE in RA.

When estimating ASCVD risk among patients with RA, calibration of PREVENT (SIR 2.25, 95% CI 2.19–2.32) was worse than that of the PCE (SIR 1.38, 95% CI 1.34–1.41) (Supplementary Table 7). Although overall discrimination was similar (PREVENT Harrel’s C-statistic 0.66 vs PCE 0.64), sensitivity of PREVENT was lower than the PCE for 10-year ASCVD risk (61.9% vs 76.0%). PREVENT reclassified ASCVD risk calculated by the PCE in 5,772 individuals with RA and exhibited a modest NRI of 5.27% (Table 3). The majority (n = 5,021, 87.0%) of these were patients reclassified as low or borderline risk who did not experience an ASCVD outcome in follow-up. Among 657 reclassified patients who experienced ASCVD in follow-up, 626 of these were inappropriately reclassified by PREVENT as low or borderline risk. In contrast, PREVENT appropriately reclassified 31 patients who experienced ASCVD as intermediate or high risk. Reclassification of ASCVD risk was similar after incorporating ADI into PREVENT (NRI 5.03%). The NRI was reduced (0.03%) when using PREVENT incorporating HBA1c. This was driven by fewer event-free patients appropriately reclassified into the low-risk category (n = 2,684, 60.6%), although fewer patients were inappropriately reclassified as low risk and experienced ASCVD in follow-up (n = 384, 8.7%).

## DISCUSSION

This is among the first, and to our knowledge the largest, study to evaluate the performance of PREVENT in predicting clinical CVD events in patients with RA, the most common systemic autoimmune arthritis burdened by excess CVD morbidity and mortality rates.^3^ Leveraging national VHA data, we found that PREVENT underestimates CVD risk in RA compared with controls and exhibits poor sensitivity for 10-year ASCVD and HF development. When compared with the PCE for ASCVD prediction in RA, PREVENT underestimated risk to a greater degree, demonstrated lower sensitivity, and reclassified a greater number of patients as low or borderline risk who subsequently experienced an ASCVD event. Our findings underscore the continued need for tailored CVD risk assessment in patients with RA and suggest that the PREVENT calculator should be used with caution in patients with RA, particularly among those with estimated low or borderline risk.

Underlying mechanisms of CVD risk in RA are multifactorial, including systemic inflammation,^30^ medications (eg, glucocorticoids) frequently used in RA,^31^ overrepresented CVD risk factors,^32,33^ lipoprotein and endothelial dysfunction,^34,35^ and immunologic effects of autoantibody formation in RA.^36–38^ As a result, CVD risk in RA has proven difficult to accurately capture using both general population and RA-specific risk calculators.^4,5,9,31,39^ In a recent study of 161 patients with RA, PREVENT exhibited a lower predictive performance than the PCE for identifying subclinical atherosclerosis using coronary artery calcium (CAC) scoring.^40^ Building upon these findings using surrogate CVD outcomes, we captured 6,170 clinical CVD events among 30,687 patients with RA, identifying an over 2-fold greater observed vs predicted ASCVD event rate and a 1.4-fold greater observed HF event rate in RA using PREVENT. Underestimation of CVD risk in RA was greatest among those with the lowest BMI and LDL values, consistent with known obesity and lipid paradoxes in RA. These findings emphasize the need for providers to recognize that weight loss, low BMI, and low LDL may result from uncontrolled RA, which in turn increases CVD risk and signifies patients that may require a lower threshold for initiation of primary CVD prevention strategies.^28,29^

A central purpose of CVD risk stratification is to aid in decisions for primary ASCVD prevention (eg, lipid-lowering agents).^41^ Pharmacotherapy is often not recommended for those at an estimated risk of <7.5% without additional risk stratification alongside shared decision-making.^41^ In ASCVD-specific models, sensitivity using a 7.5% cutoff was just 61.9% in patients with RA, lower than when using the PCE (76%). The implications of this are illustrated by 631 patients with RA developing ASCVD who were inappropriately reclassified into low or borderline risk categories using PREVENT, compared with only 31 patients appropriately reclassified as intermediate or high risk. These findings are consistent with recent National Health and Nutrition Examination Surveys data, projecting that disproportionate reclassification to lower risk categories will lead to reduced statin or antihypertensive eligibility among nearly 16 million people and account for approximately 107,000 additional ASCVD events.^42^ This is of critical concern in RA given the heightened risk of CVD, more severe underestimation of CVD risk using PREVENT, and already suboptimal use of CVD prevention strategies in these patients.^43–45^ Given the known burden of CVD morbidity and mortality rates in RA, improved decision-making tools are needed to identify low-risk patients with RA who would benefit from additional risk stratification (eg, CAC scoring) and/or initiation of primary prevention therapies.

The integrated VHA care model results in a higher frequency of HBA1c testing,^46^ facilitating the first evaluation of PREVENT incorporating HBA1c in RA. We found incorporating HBA1c, but not ADI, in PREVENT calculation led to less underestimation and increased sensitivity for ASCVD risk prediction in RA. These improvements are in line with demonstrated effects of RA-related inflammation on insulin resistance and incident diabetes^47,48^ and may shed light on the importance of metabolic dysfunction and diabetes severity as mediators of CVD risk in RA. Preferential inclusion of HBA1c values should be considered when using PREVENT for CVD risk stratification in RA, and more aggressive diabetes screening and management may be an important area for mitigating CVD risk in this population.

There are limitations to this study. The cohort was predominantly men, which reflects the VHA population and may limit generalizability of these findings; however, we had a substantial number of women (n = 43,619) to demonstrate that although calibration modestly improved in women, a greater underestimation of CVD risk in those with RA compared to non-RA controls persists. Although this differs from cohorts in which the PREVENT equations were derived, matched controls within the VHA system were used to facilitate internal comparison of risk calculator performance in patients with RA vs without RA. Similarly, the VHA population predominantly self-reports White race, which was more common among patients with RA (75.5% vs 63.1%), driven by a larger proportion of missing race information among patients without RA (16.1 vs 6.7%). However, race is not included in the PREVENT equations because it represents a social, rather than biologic construct, and inclusion of the ADI (a measure of socioeconomic deprivation) did not meaningfully impact performance of PREVENT. Although misclassification of CVD outcomes is possible in large administrative datasets, this would be expected to be nondifferential between RA cases and controls and lead to underestimates in the SIR differences seen in this analysis. As patients with RA are seen more regularly by the health care system, there is risk for detection bias toward more frequent CVD outcomes in RA compared with controls. Lastly, there was missingness observed in PREVENT calculator variables; however, results were similar in analyses leveraging multiple imputations and in complete case analyses.

In summary, we found that PREVENT underestimates risk and exhibits poor sensitivity for ASCVD and HF development in patients with RA, which was partially mitigated when incorporating HBA1c values. As it demonstrated the most favorable balance of calibration and discrimination among both PCE and PREVENT calculators, our findings support the preferential use of PREVENT calculators incorporating HBA1c in patients with RA as well as more frequent HBA1c screening in line with VHA integrated care strategies. Moreover, these data highlight the need for adjusted or additional risk stratification measures to aid in identifying patients with RA with low or borderline ASCVD risk who may benefit from primary CVD prevention therapies, particularly those with low LDL or BMI in the context of active RA. Taken together, there remains a continued need for accurate CVD risk stratification strategies in RA.

## Supplementary Material

supplement revised

Additional supplementary information cited in this article can be found online in the Supporting Information section (https://acrjournals.onlinelibrary.wiley.com/doi/10.1002/art.70081).

## Figures and Tables

**Table 1.** Baseline characteristics of study cohort*

Characteristics	RA	non-RA

N	30,687	231,752
Age, mean (SD)	60.9 (10.0)	59.6 (10.1)
Male sex, n (%)	26,050 (84.9)	192,770 (83.2)
Race, n (%)		
White	23,143 (75.4)	146,331 (63.1)
Black	4,740 (15.4)	40,967 (17.7)
Other	751 (2.4)	7,102 (3.1)
Missing or unknown	2,053 (6.7)	37,352 (16.1)
BMI category, n (%),kg/m^2^		
18.5 to <25	6,001 (19.6)	34,913 (15.1)
25 to <30	12,010 (39.1)	71,855 (31.0)
30 to <35	8,826 (28.8)	51,007 (22.0)
35 to <40	3,445 (11.2)	19,431 (8.4)
Unknown or missing or invalid	405 (1.3)	54,546 (23.5)
Blood pressure, mean (SD), mm Hg		
Systolic	128.4 (17.4)	129.7 (17.1)
Diastolic	74.5 (10.3)	75.7 (10.3)
Smoking status, n (%)		
Current	16,138 (52.6)	95,126 (41.0)
Former	9,336 (30.4)	55,565 (24.0)
Never	4,677 (15.2)	47,990 (20.7)
Unknown or missing	536 (1.7)	33,071 (14.3)
Diabetes, n (%)	6,879 (22.4)	36,386 (15.7)
Hypertension, n (%)	15,707 (51.2)	81,897 (35.3)
Chronic lung disease, n (%)	4,637 (15.1)	16,929 (7.3)
Cholesterol level, mean (SD)		
Total	183.3 (34.9)	186.1 (35.7)
LDL	105.2 (33.5)	107.1 (34.5)
HDL	46.3 (14.5)	46.4 (14.2)
eGFR, mean (SD)	81.5 (19.9)	80.1 (18.7)
Medications, n (%)		
Aspirin	3,241 (10.6)	14,273 (6.2)
Lipid-lowering therapy	11,793 (38.4)	56,400 (24.3)
Antihypertensive	17,066 (55.6)	74,272 (32.0)
PREVENT risk category, n (%)		
Overall CVD		
Low or borderline (<7.5%)	12,113 (39.5)	114,785 (49.5)
Intermediate (7.5%–19.9%)	14,396 (46.9)	97,206 (41.9)
High (≥20%)	4,178 (13.6)	19,761 (8.5)
ASCVD		
Low or borderline (<7.5%)	20,674 (67.4)	175,031 (75.5)
Intermediate (7.5%–19.9%)	9,636 (31.4)	54,879 (23.7)
High (≥20%)	377 (1.2)	1,842 (0.8)
Heart failure		
Low or borderline (<7.5%)	20,842 (67.9)	177,788 (76.7)
Intermediate (7.5%–19.9%)	8,718 (28.4)	49,129 (21.2)
High (≥20%)	1,127 (3.7)	4,835 (2.1)

* Values are those before imputing missing values. ASCVD, atherosclerotic cardiovascular disease; BMI, body mass index; CVD, cardiovascular disease; eGFR, estimated glomerular filtration rate; HDL, high-density lipoprotein; LDL, low-density lipoprotein; PREVENT, Predicting Risk of Cardiovascular Disease Events; RA, rheumatoid arthritis.

**Table 2.** Calibration and discrimination of the Predicting Risk of Cardiovascular Disease Events calculators in rheumatoid arthritis*

Cardiovascular Outcome	Standard calculator		Including ADI		Including hemoglobin A1c	
	RA	Non-RA	RA	Non-RA	RA	Non-RA

Overall CVD						
SIR	1.83	1.44	1.87	1.48	1.21	0.88
Sensitivity, %	85.7	83.2	85.2	82.1	93.1	94.9
Harrel’s C-statistic	0.67	0.70	0.67	0.71	0.68	0.70
ASCVD						
SIR	2.25	1.86	2.31	1.91	1.45	1.11
Sensitivity, %	61.9	55.2	60.0	53.2	80.3	80.1
Harrel’s C-statistic	0.66	0.70	0.66	0.70	0.67	0.70
Heart failure						
SIR	1.41	0.89	1.88	1.18	0.79	0.46
Sensitivity, %	63.2	57.4	45.3	38.6	79.9	81.0
Harrel’s C-statistic	0.69	0.71	0.67	0.69	0.69	0.71

* Standardized incidence ratios calculated as the ratio of observed CVD event rates to predicted CVD event rates. Values >1 indicate underestimation, whereas values <1 indicate overestimation by the risk calculator. Sensitivity calculated based on a predicted risk cutoff of 7.5% capturing those who experience CVD events. ADI, area deprivation index; ASCVD, atherosclerotic cardiovascular disease; CVD, cardiovascular disease; RA, rheumatoid arthritis; SIR, standardized incidence ratio.

**Table 3.** Reclassification of predicted ASCVD risk in patients with rheumatoid arthritis using the PREVENT calculator compared with the Pooled Cohort Equation*

Reclassified category	Experienced ASCVD, n^a^	ASCVD-free, n^a^	Net reclassification index, % (95% CI)

PREVENT			
≥7.5%	31	94	5.27 (3.94 to 6.68)
<7.5%	626	5,021	
PREVENT (plus ADI)			
≥7.5%	29	101	5.03 (−0.77 to 6.34)
<7.5%	674	5,264	
PREVENT (plus HBA1c)			
≥7.5%	140	1,223	0.03 (−0.87 to 6.26)
<7.5%	384	2,684	

* ADI, area deprivation index; ASCVD, atherosclerotic cardiovascular disease; CI, confidence interval; HBA1C, hemoglobin A1c; PREVENT, Predicting Risk of Cardiovascular Disease Events.

a Values reflect the number of patients experiencing or not experiencing the ASCVD outcome who were reclassified up into the intermediate or high-risk PREVENT category (≥7.5%) or reclassified down into the low-risk category (<7.5%).
