# Supplementary material for: Performance of the Predicting Risk of Cardiovascular Disease Events Calculator in Rheumatoid Arthritis
Source: Arthritis Rheumatol. Author manuscript; Available in PMC 2026 May 7. (PMC13148063; doi:10.1002/art.70081)
Supplement: Supplementary file 1 [file NIHMS2159480-supplement-supplement_revised.docx]

**Supplementary Material**

Title: Performance of the Predicting Risk of Cardiovascular Disease EVENTs Calculator in Rheumatoid Arthritis 

Johnson TM, et al.

Supplementary Table 1. Variables included in the Predicting Risk of risk of cardiovascular EVENTs (PREVENT) and Pooled Cohort Equations (PCE) Calculators

Supplementary Table 2. Cardiovascular disease event rates in rheumatoid arthritis and matched controls outcome 

Supplementary Table 3. Calibration and discrimination of the Predicting Risk of cardiovascular EVENTs calculators stratified by Seropositivity of Rheumatoid Arthritis Cases

Supplementary Table 4. Sensitivity analyses examining calibration and discrimination of the Predicting Risk of cardiovascular EVENTs calculators

Supplementary Table 5. Calibration and discrimination of the Predicting Risk of cardiovascular EVENTs calculators stratified by Sex

Supplementary Table 6. Calibration and discrimination of the Predicting Risk of cardiovascular EVENTs calculators compared with the Pooled Cohort Equation for ASCVD prediction in patients with rheumatoid arthritis.

| **Supplementary Table** **1.** Variables included in the Predicting Risk of risk of cardiovascular EVENTs (PREVENT) and Pooled Cohort Equations (PCE) Calculators |  |  |  |
| --- | --- | --- | --- |
| **Variable** | **PREVENT** | **PCE** | **Source** |
| Age | X | X | VHA enrollment records |
| Sex | X | X | VHA enrollment records |
| Race |  | X | VHA enrollment records |
| BMI | X |  | Vital sign data from CDW |
| Lipid levels |  |  |  |
| Total | X | X | Laboratory data from CDW |
| HDL | X | X | Laboratory data from CDW |
| LDL |  | X | Laboratory data from CDW |
| Systolic BP | X | X | Vital sign data from CDW |
| Diastolic BP |  | X | Vital sign data from CDW |
| Diabetes | X | X | Diagnostic (ICD-9/10) codes or laboratory data from CDW |
| Smoking status | X | X | VHA Health Factors |
| Antihypertensive | X | X | Pharmacy dispensing data from CDW |
| Lipid lowering therapy | X | X | Pharmacy dispensing data from CDW |
| Aspirin |  | X | Pharmacy dispensing data from CDW |
| eGFR | X |  | Laboratory data from CDW |
| Hemoglobin A1c | (optional) |  | Laboratory data from CDW |
| ADI | (optional) |  | Zip code data from CDW |
| Abbreviations: ADI, area of deprivation index; CDW, Corporate Data Warehouse; eGFR, estimated glomerular filtration rate; BMI, body mass index; BP, blood pressure; HDL, high density lipoprotein; LDL, low density lipoprotein; VHA, Veteran’s Health Administration |  |  |  |

| **Supplementary Table** **2.** Cardiovascular disease event rates in rheumatoid arthritis and matched controls |  |  |
| --- | --- | --- |
|  | **RA (n=30,687)** | **Non-RA (n=231,752)** |
| **Overall CVD** ^**a**^ |  |  |
| N events | 6,170 | 30,679 |
| Follow up time (PY) | 232,898 | 1,830,638 |
| Incidence rate (95% CI), per 1000 PY | 24.95 (24.33-25.58) | 16.20 (16.0-16.38) |
| **ASCVD** ^**b**^ |  |  |
| N events | 4,413 | 23,648 |
| Follow up time (PY) | 238,047 | 1,848,317 |
| Incidence rate (95% CI), per 1000 PY | 17.84 (17.32-18.38) | 12.49 (12.33-12.65) |
| **Heart Failure** |  |  |
| N events | 2,848 | 11,003 |
| Follow up time (PY) | 241,558 | 1,873,728 |
| Incidence rate (95% CI), per 1000 PY | 11.52 (11.10-11.95) | 5.81 (5.70-5.92) |
| ^a ^Overall CVD is a composite of fatal and non-fatal myocardial infarction, stroke, and heart failure ^b^ ASCVD is a composite of fatal and non-fatal myocardial infarction and stroke Abbreviations: ASCVD, atherosclerotic cardiovascular disease; PY, person-years; RA, rheumatoid arthritis; |  |  |

| **Supplementary Table** **3.** Calibration and discrimination of the Predicting Risk of cardiovascular EVENTs calculators stratified by Seropositivity of Rheumatoid Arthritis Cases |  |  |  |  |
| --- | --- | --- | --- | --- |
|  | **Seropositive RA** **(n=20,006)** | **Non-RA**^**a**^ **(n=151,994)** | **Seronegative RA** **(n=8,625)** | **Non-RA**^**b**^ **(n=64,414)** |
| Overall CVD |  |  |  |  |
| *SIR (95% CI)* | 1.88 (1.83-1.94) | 1.46 (1.44-1.48) | 1.70 (1.62-1.79) | 1.40 (1.37-1.43) |
| *Sensitivity, %* | 85.4 | 82.8 | 85.3 | 83.1 |
| *Harrel’s C statistic* | 0.67 | 0.70 | 0.68 | 0.72 |
| ASCVD |  |  |  |  |
| *SIR (95% CI)* | 2.31 (2.22-2.39) | 1.81 (1.76-1.85) | 2.16 (2.04-2.29) | 1.81 (1.76-1.85) |
| *Sensitivity, %* | 61.3 | 53.9 | 62.6 | 56.5 |
| *Harrel’s C statistic* | 0.66 | 0.70 | 0.68 | 0.72 |
| Heart Failure |  |  |  |  |
| *SIR (95% CI)* | 1.49 (1.42-1.56) | 0.90 (0.88-0.92) | 1.33 (1.23-1.43) | 0.85 (0.81-0.88) |
| *Sensitivity, %* | 61.4 | 56.4 | 66.2 | 58.6 |
| *Harrel’s C statistic* | 0.68 | 0.71 | 0.70 | 0.72 |
| ^a ^Non-RA controls matched to patients with seropositive RA ^b ^Non-RA controls matched to patients with seronegative RA Abbreviations: ASCVD, atherosclerotic cardiovascular disease; CI, confidence interval; CVD, cardiovascular disease; RA, rheumatoid arthritis; SIR, standardized incidence ratio |  |  |  |  |

| **Supplementary Table 4.** Sensitivity analyses examining calibration and discrimination of the Predicting Risk of cardiovascular EVENTs calculators |  |  |
| --- | --- | --- |
|  | **RA** | **Non-RA** |
| **Restricting to age 65 and older (n=10,352 RA; n=65,687 non-RA)** |  |  |
| Overall CVD |  |  |
| *SIR (95% CI)* ^*a*^ | 1.64 (1.58-1.70) | 1.32 (1.29-1.34) |
| *Sensitivity, %* | 96.9 | 98.0 |
| *Harrel’s C statistic* | 0.60 | 0.61 |
| ASCVD |  |  |
| *SIR (95% CI)* ^*a*^ | 2.07 (1.98-2.16) | 1.78 (1.75-1.82) |
| *Sensitivity, %* | 85.8 | 80.6 |
| *Harrel’s C statistic* | 0.59 | 0.60 |
| Heart Failure |  |  |
| *SIR (95% CI)* ^*a*^ | 1.18 (1.11-1.24) | 0.69 (0.67-0.71) |
| *Sensitivity, %* | 90.0 | 86.2 |
| *Harrel’s C statistic* | 0.64 | 0.66 |
| **Complete Case Analysis (n=21,362 RA; n=64,783 non-RA)** |  |  |
| Overall CVD |  |  |
| *SIR (95% CI)* ^*a*^ | 1.70 (1.65-1.75) | 1.34 (1.31-1.37) |
| *Sensitivity, %* | 89.4 | 90.9 |
| *Harrel’s C statistic* | 0.69 | 0.69 |
| ASCVD |  |  |
| *SIR (95% CI)* ^*a*^ | 2.09 (2.01-2.16) | 1.68 (1.65-1.72) |
| *Sensitivity, %* | 67.6 | 67.7 |
| *Harrel’s C statistic* | 0.68 | 0.68 |
| Heart Failure |  |  |
| *SIR (95% CI)* ^*a*^ | 1.31 (1.25-1.37) | 0.89 (0.86-0.89) |
| *Sensitivity, %* | 66.8 | 69.3 |
| *Harrel’s C statistic* | 0.70 | 0.71 |
| ^a ^Standardized incidence ratios calculated as the ratio of observed CVD event rates to predicted CVD event rates. Sensitivity calculated based on a predicted risk cutoff of 7.5%. Abbreviations: ASCVD, atherosclerotic cardiovascular disease; CI, confidence interval; CVD, cardiovascular disease; RA, rheumatoid arthritis; SIR, standardized incidence ratio |  |  |

| **Supplementary Table 5.** Calibration and discrimination of the Predicting Risk of cardiovascular EVENTs calculators stratified by Sex |  |  |
| --- | --- | --- |
|  | **RA** | **Non-RA** |
| **Female (4,637=RA; 38,982=non-RA)** |  |  |
| Overall CVD |  |  |
| *SIR (95% CI)* | 1.44 (1.29-1.59) | 0.98 (0.94-1.03) |
| *Sensitivity, %* | 60.7 | 59.9 |
| *Harrel’s C statistic* | 0.74 | 0.78 |
| ASCVD |  |  |
| *SIR (95% CI)* | 1.65 (1.45-1.87) | 1.19 (1.12-1.25) |
| *Sensitivity, %* | 35.0 | 32.6 |
| *Harrel’s C statistic* | 0.73 | 0.78 |
| Heart Failure |  |  |
| *SIR (95% CI)* | 1.20 (1.03-1.39) | 0.71 (0.66-0.77) |
| *Sensitivity, %* | 38.01 | 37.22 |
| *Harrel’s C statistic* | 0.74 | 0.78 |
| **Male (26,050=RA; 192,770=non-RA)** |  |  |
| Overall CVD |  |  |
| *SIR (95% CI)* | 1.87 (1.82-1.91) | 1.48 (1.47-1.50) |
| *Sensitivity, %* | 87.3 | 84.6 |
| *Harrel’s C statistic* | 0.65 | 0.68 |
| ASCVD |  |  |
| *SIR (95% CI)* | 2.30 (2.23-2.37) | 1.92 (1.89-1.95) |
| *Sensitivity, %* | 63.5 | 56.4 |
| *Harrel’s C statistic* | 0.64 | 0.67 |
| Heart Failure |  |  |
| *SIR (95% CI)* | 1.42 (1.37-1.48) | 0.90 (0.88-0.92) |
| *Sensitivity, %* | 64.9 | 58.7 |
| *Harrel’s C statistic* | 0.67 | 0.69 |
| Abbreviations: ASCVD, atherosclerotic cardiovascular disease; CI, confidence interval; CVD, cardiovascular disease; RA, rheumatoid arthritis; SIR, standardized incidence ratio |  |  |

| **Supplementary Table 6.** Calibration and discrimination of the Predicting Risk of cardiovascular EVENTs calculators stratified by diabetes diagnoses |  |  |
| --- | --- | --- |
|  | **RA** | **Non-RA** |
| **Diabetes (RA = 6,879; Non-RA = 9,316)** |  |  |
| Overall CVD |  |  |
| *SIR (95% CI)* ^*a*^ | 1.72 (1.65-1.80) | 1.40 (1.34-1.46) |
| *Sensitivity, %* | 95.0 | 97.6 |
| *Harrel’s C statistic* | 0.62 | 0.61 |
| ASCVD |  |  |
| *SIR (95% CI)* ^*a*^ | 2.10 (1.99-2.21) | 1.79 (1.71-1.88) |
| *Sensitivity, %* | 85.6 | 87.9 |
| *Harrel’s C statistic* | 0.62 | 0.60 |
| Heart Failure |  |  |
| *SIR (95% CI)* ^*a*^ | 1.31 (1.24-1.40) | 0.89 (0.83-0.95) |
| *Sensitivity, %* | 89.5 | 90.8 |
| *Harrel’s C statistic* | 0.62 | 0.63 |
| **No Diabetes (RA = 23,808 ; Non-RA = 155,967)** |  |  |
| Overall CVD |  |  |
| *SIR (95% CI)* ^*a*^ | 1.89 (1.83-1.95) | 1.45 (1.43-1.48) |
| *Sensitivity, %* | 81.2 | 76.0 |
| *Harrel’s C statistic* | 0.67 | 0.70 |
| ASCVD |  |  |
| *SIR (95% CI)* ^*a*^ | 2.33 (2.25-2.42) | 1.89 (1.86-1.92) |
| *Sensitivity, %* | 50.7 | 42.0 |
| *Harrel’s C statistic* | 0.66 | 0.70 |
| Heart Failure |  |  |
| *SIR (95% CI)* ^*a*^ | 1.47 (1.40-1.54) | 0.87 (0.85-0.90) |
| *Sensitivity, %* | 48.1 | 38.6 |
| *Harrel’s C statistic* | 0.68 | 0.69 |
| Abbreviations: ASCVD, atherosclerotic cardiovascular disease; CI, confidence interval; CVD, cardiovascular disease; RA, rheumatoid arthritis; SIR, standardized incidence ratio |  |  |

| **Supplementary Table 7.** Calibration and discrimination of the Predicting Risk of cardiovascular EVENTs calculators compared with the Pooled Cohort Equation for ASCVD prediction in patients with rheumatoid arthritis. |  |  |
| --- | --- | --- |
|  | **PREVENT** | **PCE** |
| SIR (95% CI) ^a^ | 2.25 (2.19-2.32) | 1.38 (1.34-1.41) |
| Sensitivity, % | 61.9 | 76.0 |
| Harrel’s C statistic | 0.66 | 0.64 |
| ^a ^Standardized incidence ratios calculated as the ratio of observed MACE event rates to predicted MACE event rates. Sensitivity calculated based on a predicted risk cutoff of 7.5%. Abbreviations: ASCVD, atherosclerotic cardiovascular disease; CI, confidence interval; SIR, standardized incidence ratio |  |  |
